# Acute effects of different external compression with blood flow restriction on force-velocity profile during squat and bench press exercises

**DOI:** 10.5114/biolsport.2023.112093

**Published:** 2022-03-16

**Authors:** Josep M. Serrano-Ramon, Juan M. Cortell-Tormo, Iker Bautista, Miguel García-Jaén, Iván Chulvi-Medrano

**Affiliations:** 1 Department of General and Specific Didactics, University of Alicante, Alicante, Alicante, Spain; 2 Physiotherapy, Catholic University of Valencia, Valencia, Valencia, Spain; 3 Department of Physical and Sports Education, University of Valencia, 46010 Valencia, Spain

**Keywords:** Strength, Resistance training, Occlusion, Sport, Compression, Pow

## Abstract

The aim was to compare the acute effects of bench press (BP) and squat (SQ) exercises with blood flow restriction (BFR) (40%, 60%, 80% and 100% of the complete arterial occlusion pressure (AOP)) and without BFR (CON) on the mean propulsive (Vel_MED_) and maximum (Vel_MAX_) bar velocity. Fourteen healthy, physically active males (age, 23.6 ± 4.1 years; height, 1.85 ± 0.11 m; body weight 85.4 ± 4.1 kg) took part in the study. There was one set for each testing condition (CON, 40%, 60%, 80% and 100%) with 6 repetitions for BP and 6 repetitions for SQ, at 60% of 1RM, and 3 minutes of recovery between sets. The results showed statistically significant differences of the sets with 80% BFR vs. CON (mean difference [MD] = 0.035 m · s^-1^, p < 0.05, ES = 0.52 [1.02–0.03]) and 100% BFR sets vs. CON (MD = 0.074, p < 0.001, ES = 1.08 [1.79–0.38]) for BP. In the SQ exercise, statistically significant differences were found between 100% BFR vs. CON (DM = 0.031 m · s^-1^, p < 0.05), vs. 100% BFR 40% (MD = 0.04 m · s^-1^, p < 0.05). Trend analysis showed a statistically significant linear trend (F[1,9] = 34.9, p < 0.001, F[1,13] = 27.32, p < 0.001) for the Vel_MED_ in relation to the different levels of BFR. In conclusion, our results showed that BFR levels above ˜80% AOP (BP) and ˜100% AOP (SQ) produce a Vel_MED_ improvement at 60% 1RM.

## INTRODUCTION

Resistance training (RT) is a useful tool for increasing athletic performance as well as improving health [1, 2]. Traditionally, to improve muscle mass and strength capacity, a range of intensities within 60–85% of one-repetition maximum (1RM) has been recommended [3]. However, this methodology has shown remarkable evolution in recent years. New training methods and technologies such as electrostimulation [4], isoinertial methods [5] mechanical vibrations [6, 7], blood flow restriction (BFR) [8], and different combinations [9] have been a recent subject of research in the specific research literature [10].

The interest in BFR training has increased during the last five years. This kind of training methodology can provide an effective alternative to conventional training [11]. This methodology requires the application of a cuff on the proximal part of the limb [12]. The cuff should generate an external occlusion leading to BFR, which is the basis of this methodology. Therefore, of all variables that configure the BFR, the level of occlusion must be properly controlled. In this regard, the arterial occlusion pressure (AOP) has been suggested as the most precise way to prescribe the level of pressure in BFR [13]. Several pieces of research have shown that the use of AOP can be combined with RT stimuli to cause gains in strength and hypertrophy [14]. The AOP-based RT training methodology (AOP–RT) has become popular in recent years due to its potential to improve strength [11] and muscle hypertrophy [12] by applying light loads (≈20–40% of 1RM). The key point of this type of methodology is that the results obtained show similar gains compared to traditional training with medium/high loads of 60–85% 1RM [11]. This feature offers interesting practical applications in cases where RT with high loads is contraindicated (i.e., older people, patients with chronic diseases, or in recovery processes for musculoskeletal pathologies) [15].

Although the application of this BFR-RT methodology within athletic performance could yield performance benefits in many disciplines [16, 17], the latest trends in RT include the advice not to work in ranges close to full fatigue [18]. In this regard, this stresses the importance of implementing velocity as a key variable for measuring the intensity during training [19, 20]. RT training for improving sports performance is closely related to the velocity at which the load has been performed [21]. The velocity has traditionally been analysed globally although, for greater precision, it should be broken down into 3 areas: 1) mean velocity, 2) mean propulsive velocity (Vel_MED_), and 3) maximum velocity (Vel_MAX_) [22]. The magnitude of mean propulsive velocity (Vel_MED_) determines objectively the functional capacity of a subject [23], their performance level, and even their level of fatigue [24]. In addition, it contributes to optimization of the instantaneous load and avoidance of overtraining [23]. In this sense, it seems appropriate to train with multi-joint exercises for the RT [25] without neglecting the production of Vel_MED_ in addition to providing large increases in power, with a high degree of transfer to sports movements [26].

The muscle performance is determined by its contractile capacity and neuromuscular activation [27]. In BFR training, it has been shown to acutely affect muscle activation and neuromuscular fatigue [28]. These changes produce mechanical muscle tension that increases the force of muscle contractions [29]. This variation in muscle tissue produces an increase in metabolic stress and the sensitivity of the intracellular anabolic and catabolic pathways [30]. The increased sensitivity leads to the faster synthesis of muscle proteins [29]. Recently, Wilk et al. [31] studied the acute effects of external compression with BFR at 100% and 150% of AOP on maximal strength and strength-endurance performance during bench press exercise (BP) in healthy strength-trained men. Results obtained from this study revealed an increase of maximal strength with high external compression (i.e., 150% vs. without BFR, effect size [ES] = 0.19). The number of repetitions and time under tension were also increased significantly when subjects were tested at 150% of BFR. For this reason, the authors concluded that maximal strength as well as endurance performance was increased when high external compression was applied. In this line, Wilk et al. [32] found that type of cuffs (i.e., wide 96 × 13 cm vs. narrow 57 × 9 cm) during BFR had an acute effect on bar velocity and power output during BP exercise at 70% 1RM and using 90% of arterial occlusion. The authors found an increased in peak bar velocity (i.e., 18%, ES = 1.65), mean bar velocity (i.e., 13%, ES = 1.00) and peak power output (i.e., 15%, ES = 1.07) when wide BFR was compared to narrow BFR, and without BFR [33, 34]. Therefore, type of cuffs used during BFR had an influence on velocity and power in BP exercise. The authors speculate on the possibility of a “rebound effect” that would explain the increase in the peak velocity, not the average. This effect is attributed to the mechanical energy generated by the cuff, and therefore the width of the cuff would be an influential factor [34].

A recent study performed by Wilk et al. [32] showed an acute effect of type of cuffs on bar velocity in BP exercise, but no studies have investigated the acute effect of different pressure levels on bar velocity in BP and squat (SQ) exercises. Therefore, the main objective of this study was to analyse the acute effect of different degrees of partial occlusion in the arms and legs (without occlusion, 40%, 60%, 80% and 100% of AOP) on bar velocity (i.e., mean velocity of propulsive phase [Vel_MED_]) in bench press and squat exercises at 60% of 1RM in healthy active subjects.

## MATERIALS AND METHODS

### Participants

A total of 14 males took part in this study. All participants were healthy and physically active. The mean and standard deviation (± SD) of age, height, and body mass were 23.6 ± 4.1 years, 1.85 ± 0.11 m, and 85.4 ± 4.1 kg, respectively. The 1RM values were 79.33 ± 14.53 kg and 76.11 ± 6.95 kg, with a relative strength index [35] of 1.07 ± 0.003 and 1.12 ± 0.04 for BP and SQ exercises respectively. All study participants reported previous recreational experience in external resistance training for 1 to 3 years. Prior to conducting the experiment, all participants received an explanation of the purpose of the study, the evaluation procedures, and the potential risks of participating in it. Once informed, all participants signed informed consent. The experimental procedure of this investigation complied with the ethical principles of the Declaration of Helsinki. This study was approved by the University Ethics Committee (UA / 2018–11–15).

### Experimental setting

**FIG. 1:**
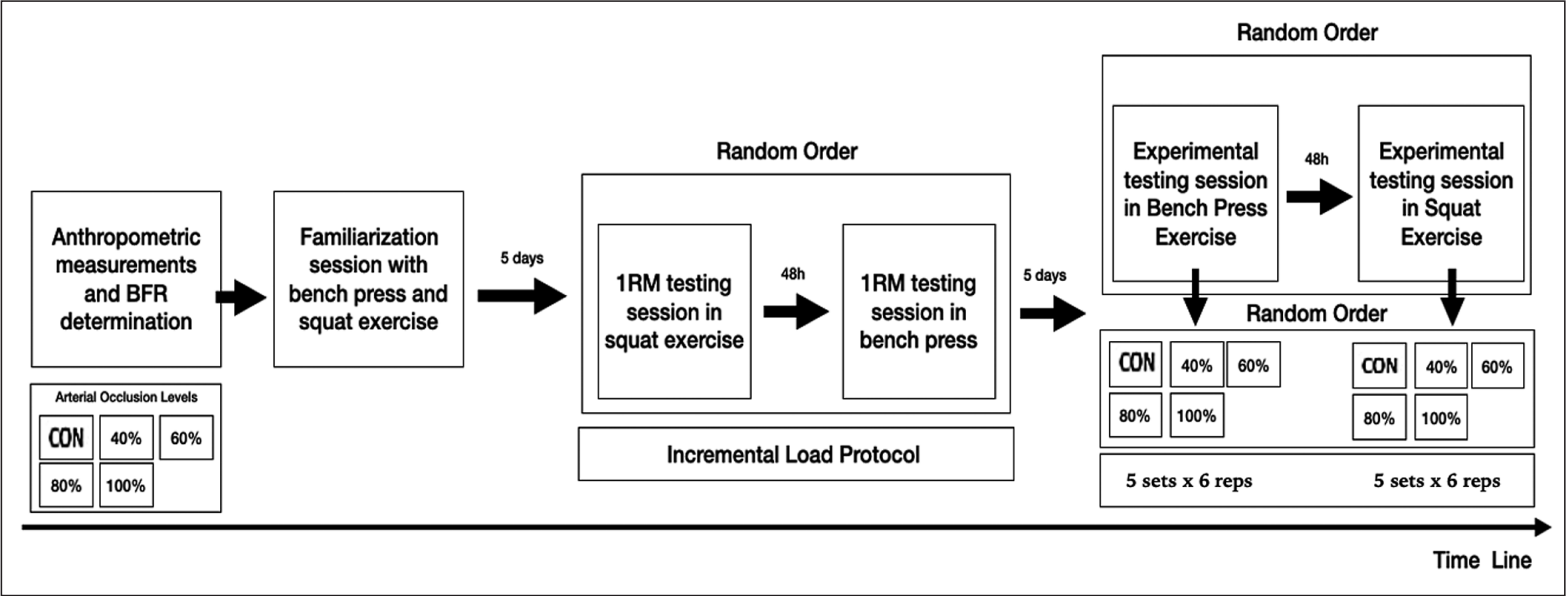
Graphic representation of the experimental design. BFR: blood flow restriction. CON: without blood flow restriction. 1RM: 1 repetition maximum.

All subjects were recruited for a total of 6 visits to the laboratory. The intervention protocol is described in Figure 1. During the first visit, informed consent was signed, and subsequently, anthropometric measurements corresponding to body mass (Avery Ltd Model 3396 ABV) and height (Holtain Ltd., Dyfed, Wales) were made. In addition, the level of partial occlusion of the venous return was individually determined for the upper and lower body. On the second visit, the participants performed a familiarization session with BP and SQ exercises. The familiarization session consisted of standardized warm-up (i.e., 10 minutes of continuous running) arm and hip mobility exercises.

### Determination of arterial occlusion pressure (AOP)

For AOP measurement, an ultrasound machine with an echo-Doppler probe was used (Doppler Sonosite Titan ultrasound machine, Sonosite, Inc., Bothwell, WA). The determination procedure took place a week before the testing procedures and was carried out by recording the flow wave next to the electrocardiographic vascular pulse scan, taking images of flow waves at the level of the inguinal and brachial artery fold [36]. Participants remained in a prone position on a stretcher during the entire procedure [37]. The pneumatic cuff (Komprimeter standard pneumatic Rudolf Riester GmbH – Bruckstr. Jungingen Germany) used for intervention was placed on the proximal portion of the arm (57 × 9 cm) and leg (96 × 13 cm) to determine 100% arterial occlusion pressure using a Doppler ultrasound scan of the femoral and brachial artery. External pressure was increasing and monitored with a manometer until the signal of blood flow disappeared in the echo-Doppler, and recorded as 100% AOP in mmHg [38]. From this value, established as 100% AOP, the relative percentages of AOP (80%, 60%, 40%) were calculated and subsequently used in the experimental protocols. Table I shows descriptive data of these relative percentage values of AOP in participants.

**TABLE 1:** Descriptive statistics of the main variables of this study. Execution velocity (m · s^-1^ ± SD) and the amount of occlusion (mmHg ± SD) of the AOP

		CON	LEVEL OF COMPRESSION FROM AOP			
			40%	60%	80%	100%
**Bench Press**	**Vel_MED_**	0.73 ± 0.06	0.77 ± 0.06	0.77 ± 0.07	0.77 ± 0.07	0.81 ± 0.05
**Exercise**	**COMPRESSION**	0	62.2 ± 6.1	93.3 ± 9.1	124.4 ± 12.1	155.5 ± 15.2

**Squat**	**Vel_MED_**	1.02 ± 0.08	1.01 ± 0.09	1.05 ± 0.08	1.04 ± 0.07	1.05 ± 0.08
**Exercise**	**COMPRESSION**	0	68.3 ± 7.7	102.4 ± 11.6	136.6 ± 15.5	170.7 ± 19.4

CON: without blood flow restriction. AOP: arterial occlusion pressure. Vel_MED_ (m · S^-1^): mean propulsive velocity in meters by seconds. mmHg: millimetres of mercury.

### Familiarization session and 1RM test

Five days prior to the beginning of the testing procedures, participants performed a preliminary familiarization session with SQ and BP with a Smith machine (Multipower Fitness Line, Peroga, Spain), which allows a constant vertical movement of the bar throughout the execution and prevents lateral movement of the bar. Two certified specialists (Certified Strength and Conditioning Specialist (NSCA-CSCS)) monitored the performance of the correct technique of both exercises. The BP exercise began in the supine position on a flat bench, with the feet resting on the floor and the hands resting on the bar slightly wider than the distance between the shoulders [39]. Bench positions and grip widths were measured to be reproduced individually for each lift. The course of the bar had to go down to the chest, just above the nipples at a controlled velocity and wait there for approximately 1 s, until the order to start the concentric phase was given. This momentary pause between the eccentric and concentric phases was applied to minimize the influence of the rebound effect and allow more consistent measurements [40]. Bouncing the bar on the chest or lifting the shoulders or torso off the bench was not allowed. In the SQ exercise the subjects started with their knees and hips fully extended forming an upright position with feet apart at shoulder height and the bar resting on the back at the level of the acromion [41]. Subjects lowered the bar until their upper thighs were below horizontal (parallel with floor) [42]. Then the reverse movement was started and the initial vertical position was ascended. In both exercises (BP and SQ) the eccentric phase was controlled and constant, but the concentric phase was carried out at maximum velocity. The execution of these exercises was always monitored by 2 experienced evaluators (NSCA-CSCS).

This familiarization session began with a warm-up, consisting of 5-minute running on a treadmill at 10 km/h and 5 min of lower limb mobilization exercises for a SQ day. After that, participants performed two sets of 8 and 6 repetitions of SQ with three minutes of rest between sets and loads of 20 and 30 kg, respectively [43]. After 48 hours, for familiarization with the BP exercise, it began with 5 min of exercise bike cycling at a light self-perceived intensity, followed by 5 min of passive stretching and mobility exercises for the upper body, for two sets of 5 repetitions with loads of 20 and 40 kg [44].

### 1 repetition maximum in squat and bench press exercises

After the standardized dynamic warm-up, an indirect and incremental test was performed to obtain the 1RM without exceeding 80% of the estimated 1RM. The 1RM allows one to know the individual load at 60% 1RM in the full squat and wide grip bench press exercise [45, 46]. The initial load was established at 20 kg (bar weight) and was progressively increased by 10 kg loads until the mean propulsive velocity (Vel_MED_) obtained was < 0.8 m · s^-1^. Then, the load was adjusted individually, with small increments (from 2.5 to 5 kg) to accurately determine 1RM at the maximum concentric velocity, with an eccentric phase that was performed at a controlled average velocity (˜ 0.57–0.77 m · s^-1^). Three repetitions were performed for light loads (≤ 50% 1RM; Vel_MED_ ≥ 0.98 m · s^-1^), two for medium loads (50–80% 1RM; Vel_MED_ ˜ 0.90–0.68 m · s^-1^), and only one repetition for the heaviest loads (> 80% 1RM; Vel_MED_ ≤ 0.68 m · s^-1^). The rest between sets ranged from 3 minutes (< 80% 1RM) to 5 minutes (> 80% 1RM), performing a complete neuromuscular recovery and receiving verbal stimuli to reach maximum velocity. The assessment device used was a linear encoder (Chronojump, Barcelona, Spain), with a sampling rate of 1.000 Hz.

### Procedures (experimental testing session)

The testing procedures were summarized in Figure 1. All experimental sessions started with a standardized warm-up identical to that used in the familiarization session. Then, each participant performed 5 sets in a randomized order, with each BFR% established (100%, 80%, 60%, 40%, and 0% –CON–) for 6 repetitions in BP and 6 repetitions in SQ exercise (48 hours after). The load was calculated at 60% of 1RM by polynomial equation [47]. For each set, a load of %1RM was used, since it is the percentage that achieves the best record in the tests, based on previous research [48–50]. This load was individually adapted through second-order polynomial [47] settings to the velocity and progressive load data during warm-up without BFR [24]. During the performance of the different sets with specific %AOP, participants were encouraged to perform each concentric repetition as quickly as they could, whereas the eccentric phase [40, 44] was performed at the controlled average velocity of < 0.80 m · s^-1^. There were 3 minutes of rest between sets, during which the BFR was not maintained. Pressure levels of each %AOP were controlled during BFR experimental intervention in each set. The test procedures were performed at the same time of day for each participant, and under the same conditions (20 ºC - 22 ºC and 55% - 65% humidity).

### Statistical analysis

All variables were expressed as mean and SD. Furthermore, all the dependent variables fulfilled the assumption of normality (i.e., Shapiro-Wilk test, p > 0.05). To analyse the influence of degrees of occlusion on execution velocity, an analysis of variance of repeated measures (RM ANOVA [5]) was performed. The degrees of occlusion had a total of 5 levels (i.e., CON, 40%, 60%, 80%, and 100%). When the sphericity assumption was not fulfilled, the degrees of freedom were corrected using the Greenhouse–Geisser approximation. The Bonferroni post hoc test was performed to analyse comparisons for each of the levels. This analysis was performed for BP and SQ exercises. The effect size (ES) was expressed using Cohen’s d. The level of significance was established at p < 0.05. All analyses were performed using statistical analysis software (SPSS Inc, Chicago, Illinois, USA).

## RESULTS

The 1RM value for BP and SQ corresponded to 79.33 ± 14.53 kg and 76.11 ± 6.95 kg, respectively. The 60% 1RM load corresponded to 47.6 ± 8.7 kg, for the bench press exercise and 45.67 ± 4.17 kg, for the squat exercise. Table I shows the descriptive statistics of the mean propulsive velocity (Vel_MED_) (m · s^-1^), and the amount of occlusion (mmHg) for all the levels of occlusion.

**FIG. 2:**
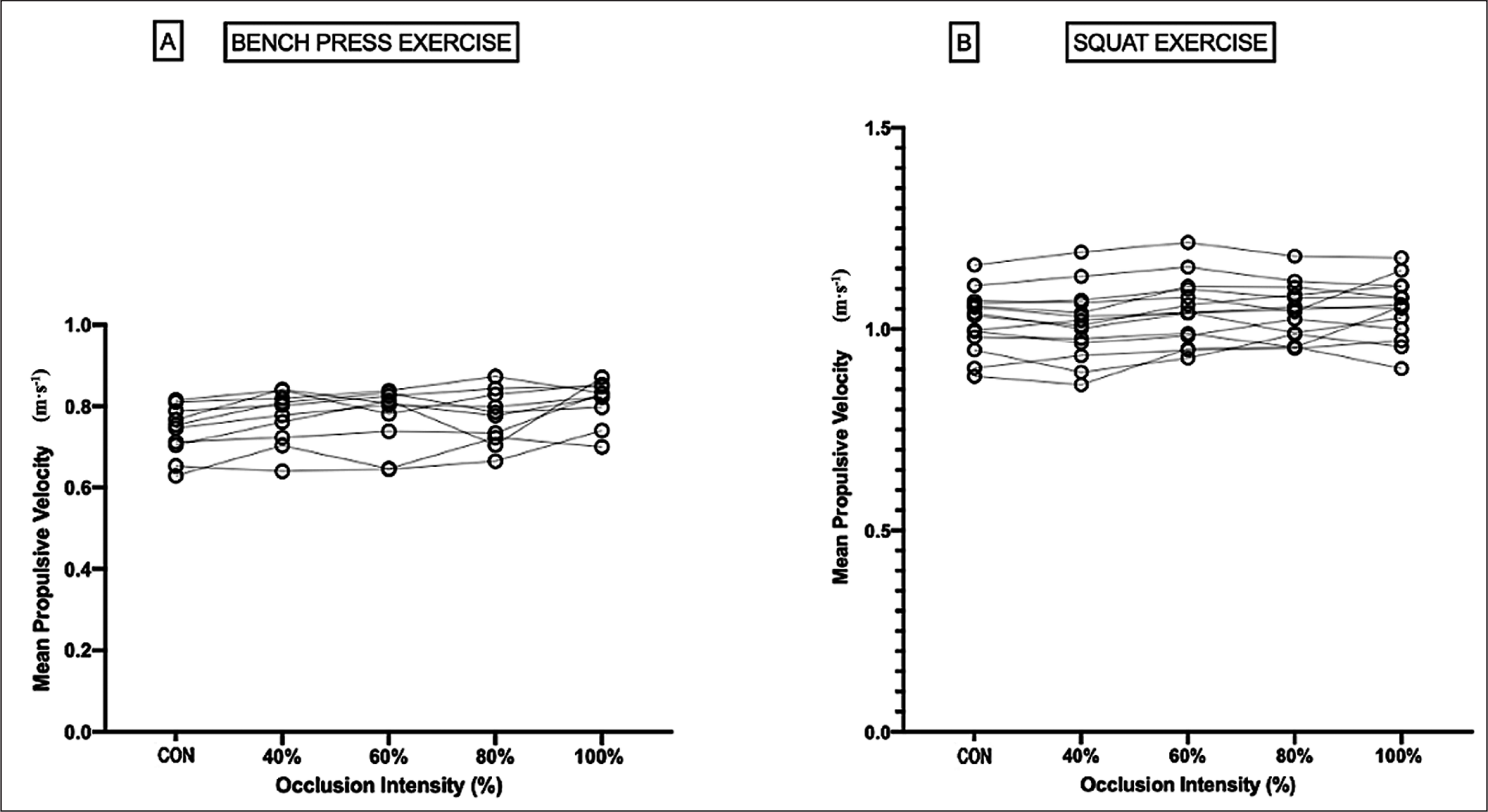
Graphical representation of the mean propulsive velocity, in relation to the levels of partial occlusion of the venous return, at 60% of the 1 maximum repetition (1RM) for the bench press (A) and squat (B) exercise.

In relation to BP exercise, RM ANOVA showed statistically significant differences (F[4,36] = 7.15, p < 0.001, h^2^_p_ = 0.44) in main effect levels of the occlusion variable. Bonferroni’s post hoc test showed statistically significant differences in the comparison of 80% BFR vs. CON (mean difference [MD] = 0.035 m · s^-1^, p < 0.05, ES = 0.52 [1.02–0.03]) and 100% BFR vs. CON (MD = 0.074 m · s^-1^, p < 0.001, ES = 1.08 [1.79–0.38]), see Figure 2A.

In SQ exercise, the RM ANOVA showed statistically significant differences (F_[2.59, 33.66]_ = 6.26, p < 0.003, h^2^_p_ = 0.33) in the main effect of the variable levels of occlusion. Bonferroni’s post hoc test showed statistically significant differences in the set comparison 100% AOP vs. CON (MD = 0.031 m · s^-1^, p < 0.05), 100% AOP vs. 40% AOP (MD = 0.04 m · s^-1^, p < 0.05), see Figure 2B.

Moreover, the trend analysis showed a statistically significant linear trend (F[1,9] = 34.9, p < 0.001, F[1,13] = 27.32, p < 0.001) for the execution velocity in relation to the different levels of occlusion (CON, 40%, 60% 80% and 100%), for the BP and SQ exercises, respectively. Finally, the percentage difference in the Vel_MED_ between the level without occlusion, and the different percentages with occlusion, were 4.57%, 4.74%, 4.78% and 10.02% for 40%, 60%, 80% and 100% of AOP, respectively.

## DISCUSSION

The main objective of this study was to analyse the acute effects of BFR at different degrees of occlusion pressure levels (i.e., CON, 40%, 60%, 80%, and 100% AOP) on the BP and SQ exercises at 60% 1RM in healthy and active men. One of the key findings was that the mean velocity of the propulsive phase at 60% 1RM was higher from 80% occlusion in comparison to CON in BP exercise and 100% occlusion in comparison to CON in SQ. In addition, the trend analysis concerning the level of occlusion described a positive linear trend, a higher level of occlusion, and a higher velocity of execution, for BP and SQ exercises.

According to our results, the level of occlusion had an acute effect on the execution velocity in BP and SQ exercises, greater effects the higher the level of occlusion. General recommendations regarding the use of BFR to improve strength and hypertrophy indicate that between 40 and 80% of AOP should be applied. Therefore, if these adaptations are the primary focus of our training, one should prior-itize training based on execution velocity. However, there are few general recommendations [29] on how to work using the BFR approach to achieve these goals. Currently, there are recent scientific studies that have studied the effects that RT-BFR can have on the velocity of execution in training with external resistance [31, 32, 33, 53].

Recently, the acute effects of the application of different levels of occlusion (i.e., WO, 100 vs 150% AOP) on multiple variables (i.e., 1RM, time under tension, number of repetitions before a load) in BP exercise were assessed [31]. This study showed that compared to the no-occlusion condition, for the 1RM assessed, the number of total repetitions at 60% 1RM and the time under tension were greater when applying a BFR of 150% AOP [31]. Moreover, the velocity of execution evaluation, (i.e., mean and peak) did not show variations throughout the different experimental conditions [31]. Wilk et al. [31] observed an acute increase in performance and suggested the possibility of a correlation between the level of external occlusion and the acute potentiation response for BP. In contrast, we found a significant positive relationship between the increase in the level of occlusion and the Vel_MED_ (see Table 1). These differences could be due to our study not having measured the 1 RM in each occlusion condition and not including reaching muscle failure. Training to muscle failure could cause a progressive loss of velocity, which increases exponentially with the number of repetitions performed [51]. In muscle failure exercises, the Vel_MED_ and Vel_MAX_ register decrease, and could occur prematurely under BFR conditions [52].

In another study, Wilk et al. [32] found large improvements in Vel_MED_ and Vel_MAX_ after BFR-RT with wide cuff (10 cm) intervention on BP. Larger increases in Vel_MED_ compared to the present study could be due to differences in the load percentage (70% vs. 60%), relative strength of the participants (1.2 vs 1), number of repetitions (3 vs. 6), and/or small differences in cuff size (10 vs. 9 cm). In contrast, in another study by Wilk et al. [33] which applied a gradual increase from 20 to 90% 1RM with 10% increments, no significant improvements in Vel_MED_ were obtained (in 60% RM) on BP. These results, contrary to the previous ones, could be due to the difference in cuff size (narrow, 4 in [33], 9 in present study and 10 cm in [32]). Regarding SQ exercise, Wilk et al. [53] found, in an incremental study from 40 to 90% 1RM, with increases of 10% of 1RM, no significant differences in Vel_MED_ at ˜80% AOP. In the present study, significant increases in Vel_MED_ were found, but at higher occlusion levels than in the Wilk et al. [53] (˜100% vs. ˜80% AOP) study. Perhaps higher levels of occlusion may be needed to influence Vel_MED_ in the lower extremities.

It has been stablished that the BFR causes physiological changes in the metabolic stress [53] that could be related to improvements in Vel_MAX_ and Vel_MED_, as a consequence of an increase in lactate concentrations and acidity (→ pH) in the intramuscular environment [54]. Furthermore, the BFR causes increased sympathetic activity and nerve impulse transmission [55] which enhances the activation of motor units, recruiting predominantly FT motor units (Fast twitch fibres) [56]. The conclusions proposed by Loenneke et al. [55] suggest that the neuromuscular effects with BFR application could have positive effects on the production of force and velocity. Another possible explanation for the positive effects of RT-BFR on Vel_MAX_ and Vel_MED_ could be related to mechanical factors associated with the width of the cuff. These advantages could be due to the mechanical work generated by the compression of the cuff and the loss of performance caused by the onset of fatigue [57]. Recent research suggests that the mechanical advantage of the compression of the cuff could increase with the application of high pressures [58]. The compression and associated effects could increase the efficiency of the mechanical joint [30] as a consequence of increased joint stability [59] and therefore allow more force to be developed [31]. The increase in the force that causes this compression is produced by an accumulation of energy in the eccentric phase that is produced in the concentric phase [34, 58, 60].

The BFR application methodology to improve sports performance has focused on guidelines for improving strength and hypertrophy [54]. Recently, it has been proven the effects of the use of BFR applied in specific movements of sports, aiming at the effects of greater specificity and transfer [61, 62, 63]. However, when aiming to train velocity-based RT-BFR it seems that it would be better to train at a load of 60–70% RM with a wide cuff and at occlusion levels above ˜80% AOP for the BP and close to ˜100% AOP for SQ. Another possible aspect to consider is that the influence of BFR on muscle activity could be modified by the characteristics of exercise [34]. For instance, differences between BP and SQ exercise could be produced by applying the cuff (BFR) on the main or another primary muscles [64]. In the BP the cuff is applied directly on the triceps brachii muscle, having less effect on the primary pectoral muscles and anterior deltoid [65]. The type of exercise performed could influence the strength and power variables in BFR conditions [65]. In addition, in the SQ exercise, the application of BFR in the lower extremities also has an effect on the main muscles [34].

Despite these findings, limitations from the current research are worthy of consideration. Only 60% of the load was analysed in relation to the force-velocity profile of each participant, and the relative strength of the participants was not established as an inclusion criterion. Future studies should analyse the influence of different occlusion conditions on ranges of upper and lower loads (i.e., > 80% 1RM and < 40% 1RM) and chronic effects of BFR on the force-velocity profile.

## CONCLUSIONS

In conclusion, our findings demonstrate that the level of AOP during BFR has a small but significant effect on the Vel_MED_ at 60% of 1RM for both BP and SQ exercises. Specifically, our results suggest that from ˜80% occlusion, Vel_MED_ improved markedly for BP exercise and ˜100% for SQ exercise, compared to sets completed without occlusion. These findings have practical application for strength and conditioning specialists because our results suggest that superimposed BFR at 80% of AOP allowed an acute neuromuscular potentiation of execution velocity.
